# Amyloid imaging for dementia in clinical practice

**DOI:** 10.1186/s12916-015-0404-6

**Published:** 2015-07-13

**Authors:** John T. O’Brien, Karl Herholz

**Affiliations:** Department of Psychiatry, University of Cambridge, Box 189, Level E4 Cambridge Biomedical Campus, Cambridge, CB2 0SP UK; The University of Manchester, Institute of Brain, Behaviour and Mental Health, Wolfson Molecular Imaging Centre, 27 Palatine Road, Manchester, M20 3LJ UK

**Keywords:** Amyloid, Dementia, Imaging, Positron emission tomography

## Abstract

In vivo imaging of brain amyloid using positron emission tomography (PET) scanning is widely used in research studies of dementia, with three amyloid PET ligands being licenced for clinical use. The main clinical use of PET is to help confirm or exclude the likely diagnosis of Alzheimer’s disease in challenging cases, where diagnostic uncertainty remains after current clinical and investigative work up. Whilst diagnostically valuable in such select cases, much wider clinical adoption, especially for very early disease, will be limited by both cost and the lack of a currently effective disease-modifying treatment that requires such early case identification. The use of amyloid imaging to appropriately stratify subjects for prognostic studies and therapeutic trials should increase the efficiency and potentially shorten the time of such studies, and its use combined with other biomarkers and genetics will likely lead to new ways of defining and classifying the dementias.

## Background

Recent advances in brain imaging have transformed the way we think about, understand, and characterise Alzheimer’s disease and other dementias. Amongst these, amyloid imaging has moved rapidly from a highly select carbon-based research tool available only in centres with cyclotrons, to full commercialisation, with three fluorinated amyloid PET tracers (florbetapir, florbetaben, and flutemetamol). These advances have allowed wider distribution, with the technique now being licenced in many countries for clinical use [1]. The ability to more directly visualise, *in vivo*, aspects of pathology in the brain, in this case amyloid deposition previously only possible at autopsy, undoubtedly represents a significant step forwards. All three amyloid imaging ligands have been tested in well-conducted, blinded studies and all demonstrate a robust correlation with brain amyloid deposition [2–4], particularly with neuritic plaques, though they also bind to amyloid elsewhere (for example, in blood vessels). It is unclear whether one imaging agent has advantages over others [5]; they are at slightly different points on the development pathway, have adopted different methods for analysing and reading scans (flutemetamol uses colour, florbetaben and florbetapir provide greyscale images) and they have slightly different tracer kinetics in terms of how scan time relates to time of injection. This commentary considers issues surrounding their likely use in clinical practice.

## Use of amyloid imaging

Amyloid imaging has been rapidly adopted by the research community, for obvious reasons, including determining when and how amyloid deposition builds up in the brain, how it relates to clinical symptoms and progression, and defining its temporal relationships with other key pathological aspects of the disease such as tau deposition, structural brain atrophy, and neuroinflammation. These questions have been the focus of several large studies which have advanced our understanding of the disease. For example, amyloid deposition appears an early event, possibly occurring up to 20 years before clinical symptoms [6]. However, nothing in clinical medicine is straight forward, and Alzheimer’s disease is no exception. So the relationship between amyloid and the other characteristic pathological hallmark of Alzheimer’s disease, tau pathology, still remains uncertain. Whilst amyloid is widely thought to be “upstream” of tau deposition, tau pathology is known to relate more closely to clinical symptoms. Whilst this fits with the hypothesis that amyloid deposition occurs first, recent studies have suggest that some people with Alzheimer’s disease may present with more tau-focused neurodegenerative change prior to evidence of substantial amyloid deposition, at least on brain imaging [7]. There remain issues to be clarified regarding how best to analyse images for research [8], as whilst for clinical reporting a “positive or negative” scan reporting approach has been adopted, which does not reflect the reality of borderline changes sometimes seen in clinical practice. Whilst such an all or none approach is arguably suitable for characterising pathological change in people with established dementia, it is unlikely to be appropriate for defining early *in vivo* changes in all situations.

In contrast to its embrace by the research community, clinical use of these ligands has been relatively limited to date, with healthcare and insurance authorities reluctant to provide clinical reimbursement because of uncertainty and debate over the perceived “added value” of amyloid imaging over current diagnostics, especially in the absence of an available disease-modifying therapy. This debate will continue while more evidence accumulates [9], though the benefits of an accurate early diagnosis are very highly valued by patients and carers and an accurate diagnosis remains at the heart of appropriate management, enshrined in the dementia plans of many countries [10]. There will be clinical situations where amyloid imaging is likely to be especially helpful. These have been highlighted through consensus groups and appropriate use criteria have been published [11]. There is an age-related increase in amyloid positivity in control subjects [12], and most likely also in patients suffering from non-Alzheimer’s diseases. Thus, supporting an Alzheimer’s diagnosis with positive amyloid imaging will be more robust in younger subjects and therefore the suggestion of its preferred use in younger subjects is not ageist, but evidence based. Some distinctions, such as between Alzheimer’s disease and frontotemporal dementia (which is not associated with amyloid deposition), are likely to be more highly informative [13], especially when presenting with progressive language impairment. It is less useful for distinguishing Alzheimer’s disease from Lewy body dementia, since the latter can also be associated with amyloid deposition [14] and other established biomarkers for this differentiation already exist [15].

However, it is likely to be a mistake to apply amyloid imaging as a diagnostic tool for our currently accepted categorical classification of disease, without accepting that this will almost certainly change over the next decade. Recent revisions of diagnostic criteria for Alzheimer’s disease propose inclusion of amyloid imaging, along with other biomarkers, to help diagnose “prodromal” disease [16], or to increase certainty of the clinical diagnosis [17]. These changes remind us that diagnostic classifications evolve with advancing knowledge. Different classification domains already exist in dementia: clinical, pathological, and genetic. They overlap but do not correlate in a one-to-one fashion, and we should not expect complete correspondence in the future. A good review on the spectrum of diseases, pathological proteins, and genetic mutations has recently been provided by Villemagne et al. [18]. Thus, demonstrations of limited correspondence between clinical diagnosis and amyloid imaging, as for instance in the recent publication by Mendez et al. [19], should not be taken as evidence for misdiagnosis by clinical or imaging assessment. It rather demonstrates that we are just at the beginning of gaining better insight into the spectrum of dementia. Of course, the most relevant classification will ultimately be one that will successfully guide therapy, and we are still struggling with that. Progress will depend on conducting a broad range of clinical trials. We should learn in that respect from oncology, where accurate multidimensional classification of disease is already a reality, mostly based on the progress of molecular analysis of blood and tissue markers. We have that possibility in the brain only to a very limited extent (via cerebrospinal fluid), making molecular imaging markers even more important. The finding of a recent failed anti-amyloid study, namely that one-third of subjects entered were amyloid PET negative to begin with [20], supports the current view that an important application of amyloid PET is the appropriate stratification of subjects for therapeutic trials.

## Conclusions

The development of amyloid imaging represents an important step change in our ability to characterise and assess patients with cognitive impairment and dementia. Currently, there are clinical situations where it promises to make an important contribution to enhancing diagnostic accuracy. However, the real advance of amyloid imaging is likely to be not just about improving diagnostics, but about appropriately selecting subjects at an early stage for disease-modifying therapies once these become available. In addition, as part of a wider biological profiling of a complex disease, it promises to drive forwards new ways of understanding and classifying the dementia.

## Abbreviations

CSF: Cerebrospinal fluid
PET: Positron emission tomography
